# Crystal structure and synthesis of the bis­(anthracene)dicuprate dianion as the dipotassium salt, [K(tetra­hydro­furan)_2_]_2_[{Cu(9,10-η^2^-anthracene)}_2_], the first anionic arene com­plex of copper

**DOI:** 10.1107/S2053229623008367

**Published:** 2023-10-03

**Authors:** Victor G. Young, William W. Brennessel, John E. Ellis

**Affiliations:** a Department of Chemistry, 207 Pleasant Street SE, University of Minnesota, Minneapolis, MN 55455, USA; b Department of Chemistry, 120 Trustee Road, University of Rochester, Rochester, NY 14627, USA; Rigaku Americas Corporation, USA

**Keywords:** anthracene, copper, arenecuprate, crystal structure, tetrahydrofuran

## Abstract

Reactions of (tri­cyclo­hexyl­phosphane)copper(I) chloride with two equivalents of potassium anthracene (KAn) in tetra­hydro­furan (THF) at 200 K provides air-sensitive but thermally stable (at 293 K) solutions from which yellow crystalline blocks of bis­[bis­(tetra­hydro­furan-κ*O*)po­tas­sium] bis­(μ-anthracene-κ^2^ *C* ^9^:*C* ^10^)di­copper, [K(THF)_2_]_2_[{Cu(9,10-η^2^-C_14_H_10_)}_2_] or [K(C_4_H_8_O)_2_]_2_[Cu_2_(C_14_H_10_)_2_], **1**, were isolated in about 50% yield. Single-crystal X-ray crystallographic analysis of **1** confirmed the presence of the first known (arene)cuprate. Also, unlike all previously known homoleptic (anthracene)metallates of *d*-block elements, which con­tain metals coordinated only to terminal rings, the organocuprate unit in **1** con­tains copper bound to the 9,10-carbons of the central ring of anthracene. No other *d*- or *f*-block metal is known to afford an anthracene or other aromatic hydro­carbon com­plex having the architecture of organodicuprate **1**.

## Abstract 2

An unprecedented example of an anionic (arene)copper com­plex has been prepared and structurally characterized in the salt [K(THF)_2_]_2_[Cu_2_(anthracene)_2_] (THF is tetra­hydro­furan), which con­tains the first homoleptic anthracene com­plex of a *d*- or *f*-block metal bound only to the central ring of anthracene.

## Introduction

Our inter­est in the stabilization of ‘naked’ atomic anions of *d*-block elements as homoleptic (arene)metallates (Ellis, 2019 ▸), where the arene is often a polycyclic aromatic hydro­car­bon or polyarene, especially naph­tha­lene or anthracene, led to an examination of ‘anionic copper’ (Rieke *et al.*, 1990 ▸) or ‘Cu^1−^’ (Stack *et al.*, 1993 ▸), reported more than 30 years ago. In these studies, the assumed, but never isolated or charac­ter­ized, cuprate species was generally prepared in tetra­hydro­furan (THF) at subambient tem­per­a­tures, *ca* 170 K, by addition of soluble Cu^I^ halide com­plexes to two equivalents of lithium naph­tha­lene (LiNp). Although the cuprate was originally speculated to be a copper analog of the mono­atomic gold anion, Au^1−^, established to be present in cesium auride, CsAu (Knecht *et al.*, 1978 ▸), and subsequently observed in single crystals of [Me_4_N][Au], wherein Au^1−^ has about the same ionic radius as Br^1−^ (Dietzel & Jansen, 2001 ▸), the *bona fide* atomic copper anion has only been identified in the gas phase (Hotop *et al.*, 1973 ▸). Indeed, to our knowledge, no sub­stance con­taining copper in a formal negative oxidation state is known in a condensed phase, possibly except for the cryo­genic species, [Cu(CO)_*n*_]^1−^ (*n* = 1, 2, or 3), which have been proposed to exist in solid neon at 4–10 K (Zhou & Andrews, 1999 ▸). Based on prior studies of com­pounds con­taining naph­tha­lene-stabilized transition-metal anions (Ellis, 2006 ▸), we believed that these highly thermolabile solutions might con­tain presently unknown homoleptic (naph­tha­lene)­cuprates (Davies, 2011 ▸). Owing to the extreme thermal instability of these solutions above 170 K, our attempts to isolate or characterize products from these reactions have failed to date. However, recognition of the usual greater thermal stability in solution and/or the solid state of (anthracene)metallates of *d*-block elements, com­pared to formally analogous (naph­tha­lene)­metallates (Kucera *et al.*, 2022 ▸), led to conducting previously unreported reactions of copper(I) halides with alkali metal anthracene radical anions, *M*An (*M* = Li, Na, or K; An = anthracene), in THF. Also, because stoichiometrically analogous naph­tha­lene and anthracene com­plexes of a given *d*-block element may possess very similar mol­ecular structures, particularly in the solid state (Ellis, 2019 ▸), identification of an (anthracene)cuprate would be of substantial inter­est in shedding light on the possible nature of the previously reported ‘anionic copper’ (Rieke *et al.*, 1990 ▸) or ‘Cu^−1^’ (Stack *et al.*, 1993 ▸). 
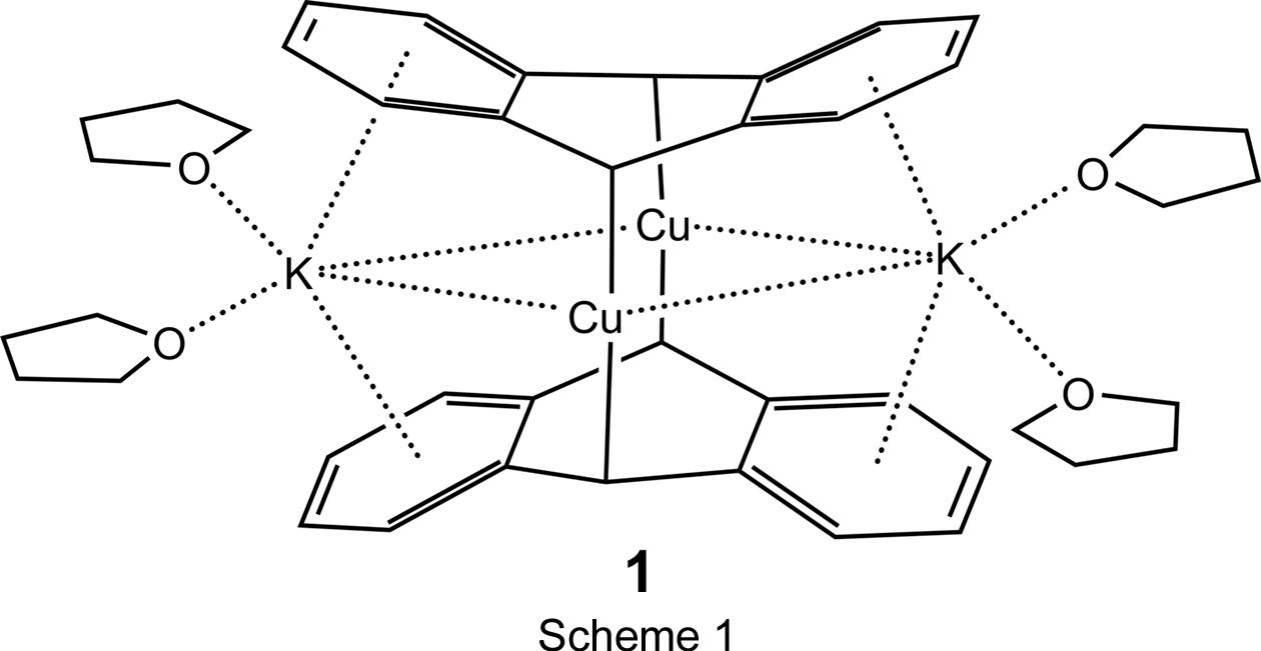


**Figure 1:**
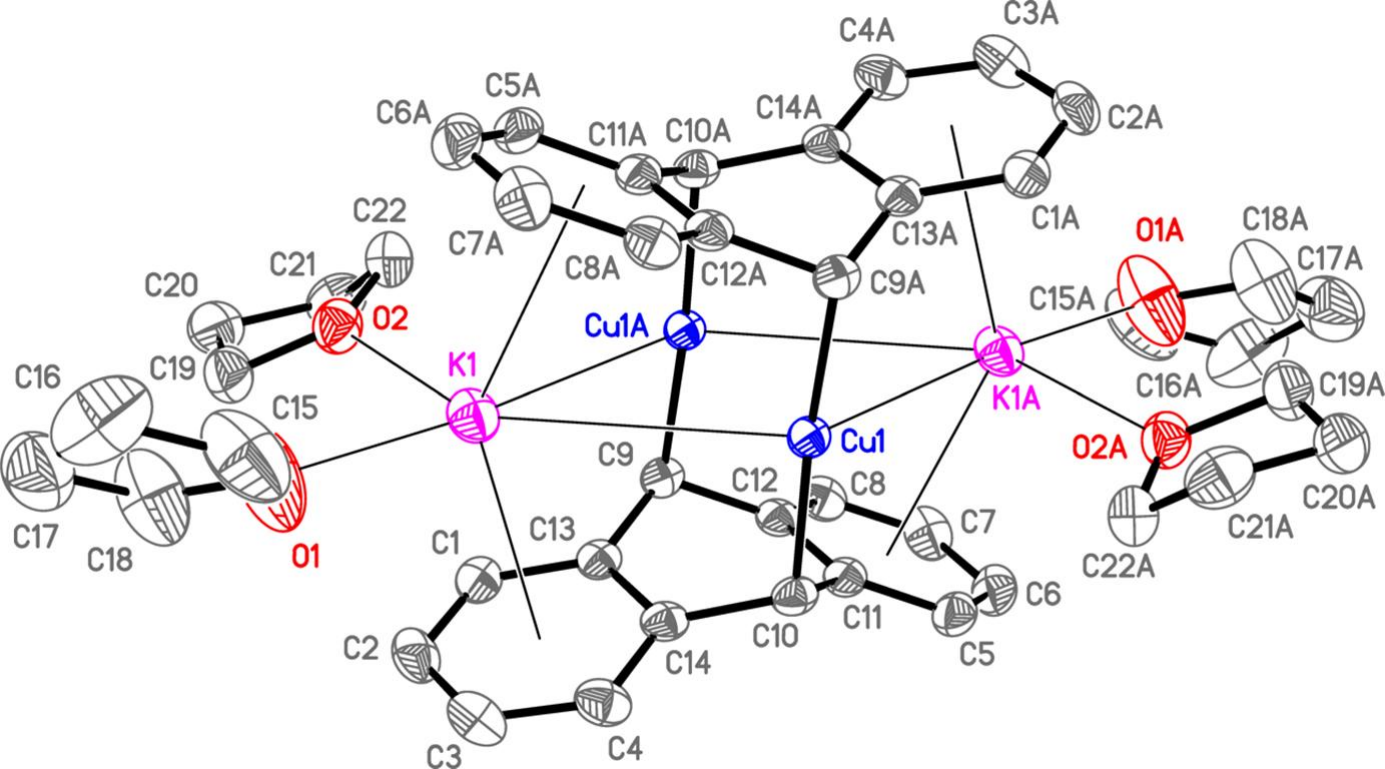
Anisotropic displacement ellipsoid plot of **1**, drawn at the 50% probability level with H atoms omitted. Symmetry-equivalent atoms (‘*A*’ label) were generated by inversion (−*x*, −*y* + 1, −*z* + 1). The K_2_Cu_2_ core is asymmetric (Å): Cu1⋯K1 = 3.6637 (15), Cu1⋯K1*A* = 3.3762 (9), and Cu1⋯Cu1*A* = 2.6172 (7).

Although ‘anionic copper’ was often prepared by the reaction of the lithium naph­tha­lene radical anion with triorganophosphane adducts of copper(I) halides, [Cu*X*(P*R*_3_)]_*n*_ (*X* = Cl, Br, or I; *R* = phenyl or *n*-but­yl) (Rieke *et al.*, 1990 ▸), owing to the particularly good solubility in THF and accessibility of [CuCl(PCy_3_)]_2_ (Cy = cyclo­hex­yl) (Churchill & Rotella, 1979 ▸), this was the only copper precursor employed in our initial study reported herein. Also, we elected not to use tri­phenyl­phosphane (PPh_3_) adducts of copper(I) halides due to the ease with which coordinated or free PPh_3_ and mixed tertiary ar­yl–alkyl phosphanes undergo reductive cleavage of P—C(ar­yl) bonds, unlike tri­alkyl­phosphanes (Chou *et al.*, 1986 ▸). Although reactions of [CuCl(PCy_3_)]_2_ with four equivalents of *M*An (*M* = Li, Na, or K) in THF appear by NMR spectra to afford similar products in solution, owing to the facile isolation and crystallization of the potassium salt, only the latter will be described now. Thus, the addition of a colorless solution of [CuCl(PCy_3_)]_2_ in THF to a dark-blue solution of KAn (molar ratio: 2 KAn/Cu) in THF at 200 K, led to the formation of an air-sensitive but thermally stable (at 293 K) yellow–brown solution, from which yellow crystalline blocks of [K(THF)_2_]_2_[{Cu(9,10-η^2^-C_14_H_10_)}_2_], **1**, were isolated (Fig. 1 ▸).

Single-crystal X-ray diffraction (SCXRD) characterization of **1** confirmed the presence of an (anthracene)cuprate, the first anionic arene com­plex of copper. However, unlike all previously known homoleptic (anthracene)metallates of the *d*-block elements (Ellis, 2019 ▸), including the recently reported bis­(anthracene)divanadate(1−) (Kucera *et al.*, 2022 ▸), which con­tain metals coordinated only to terminal rings, the organocuprate unit in **1** con­tains Cu atoms bound to the 9,10-car­bons of the central ring of anthracene. Mononuclear heteroleptic com­plexes of *d*- and *f*-block elements con­taining *M*(9,10-η^2^-anthracene) moieties have been structurally au­then­ticated for the Group 3 elements scandium (Ellis *et al.*, 2018 ▸; Ghana *et al.*, 2020 ▸; Zhu *et al.*, 2023 ▸) and lutetium (Roitershtein *et al.*, 1992 ▸, 1993 ▸), and the *f*-block elements thulium (Fedushkin *et al.*, 2001 ▸) and thorium (Yu *et al.*, 2020 ▸). Unique uranium anthracene com­plexes con­taining individual metals bound to both the terminal and central rings of different anthracene ligands have been published recently (Murillo *et al.*, 2021 ▸, 2022 ▸). Dinuclear com­plexes of scandium (Huang *et al.*, 2011 ▸, 2014 ▸), yttrium (Fryzuk *et al.*, 2000 ▸), and iron (Hatanaka *et al.*, 2012 ▸) con­taining bridging anthracenes, in which one metal binds to the terminal ring and the other *anti* to the central ring of anthracene, are also known. However, none of these species are of particular relevance to **1** and will not be discussed further. Although *d*-block com­plexes with η^6^-coordination of the central ring of untethered specially sub­sti­tuted anthracenes, for example, 2,3,6,7-tetra­meth­oxy-9,10-di­methyl­anthracene, have been confirmed recently (Karslyan *et al.*, 2017 ▸; Kuchuk *et al.*, 2019 ▸), no *d*- or *f*-block metal is known to afford an anthracene or other arene com­plex having the architecture of the organodicuprate present in **1**.

The structure of **1** (Fig. 1 ▸) consists of a contact ion-pair com­plex in which two anthracene ligands, distinctly folded about the 9,10-carbons [fold angle of 36.06 (6)°, calculated from all C atoms in the anthracene ligand] are present as centrosymmetric anthracene dianions bridging two equivalent Cu atoms in a near linear fashion across the 9,10-carbons of symmetry-related rings. Equivalent [K(THF)_2_]^1+^ counter-ions inter­act weakly with essentially planar terminal *exo*-benzene units (mean deviations from planarity of 0.011 and 0.006 Å for rings C1–C4/C14/C13 and C5–C8/C12/C11, respectively) on **1**. Details of these inter­actions will be presented in Section 3.

**Figure 2:**
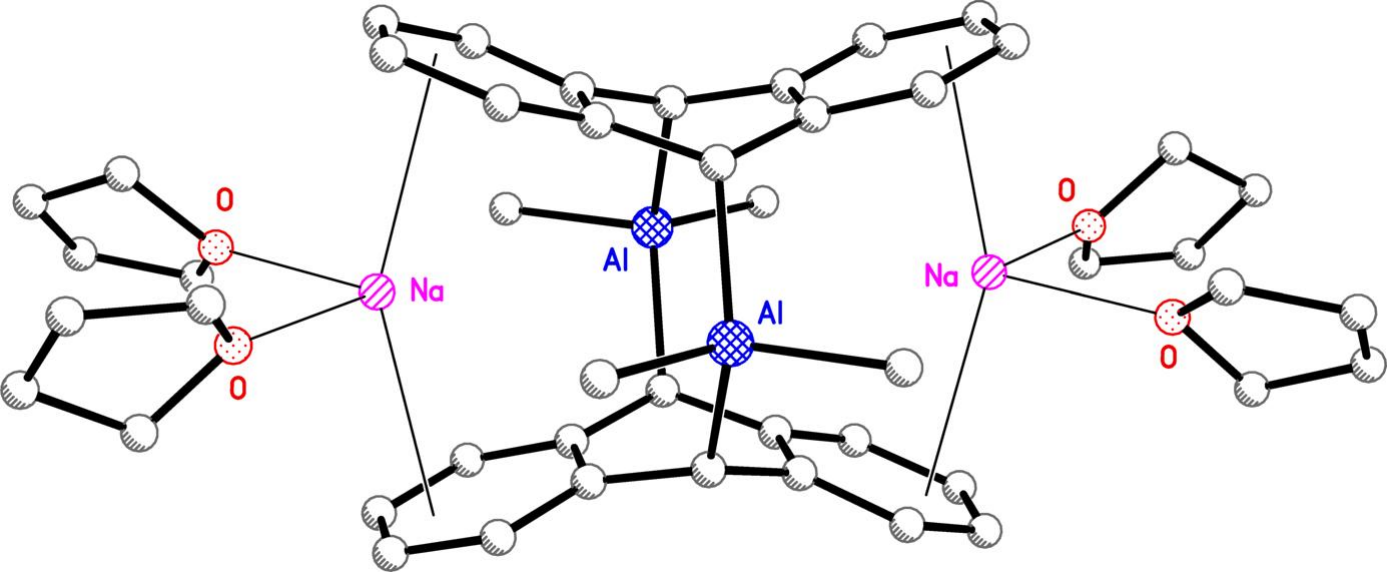
The mol­ecular plot of **3**.

To the best of our knowledge, only the long-known dimeric organoalanate com­plexes [{(Al*R*_2_)(arene)}_2_]^2−^ [*R* = methyl; arene = naph­tha­lene, **2** (Brauer & Stucky, 1970 ▸), and anthracene, **3** (see Fig. 2 ▸; Brauer & Stucky, 1972 ▸)] possess mol­ecular structures in the solid state very similar to that observed for the organocuprate dimer in **1**, *vide infra*. In particular, the coordinated anthracenes in both the cuprate, **1**, and the alanate, **3**, are effectively functioning as 9,10-di­hydro-9,10-anthrylene dianionic units with about one negative charge on each of the mixed *sp* ^2^/*sp* ^3^-hybridized 9,10 bridgehead carbons. Thus, the respective copper and aluminium anthracene com­plexes con­tain formally Cu^1+^ and Al^3+^, both closed-shell ions. One major difference in the mol­ecular structures of the anionic com­ponents of **1**, **2**, and **3** is the coordination environment about the metals. Whilst the four-coordinated Al atoms in **2** and **3** adopt distorted tetra­hedral geometries, the dimeric structure observed for organocuprate **1** con­tains identical two-coordinate Cu atoms with a nearly linear C—Cu—C angle of 174.74 (7)°. The substanti­ally dif­ferent steric impacts of the di­methyl­aluminium groups in **3** com­pared to the Cu atoms in **1** are responsible for some dramatic and surprising differences in the mol­ecular structures of these two stoichiometrically similar contact ion-pair com­plexes (see Section 3). It should also be pointed out that dimeric neutral nonmetal or metalloid adducts of anthracene are known, which have mol­ecular structures similar to the dimeric anion in **3**, including anthracene-based macrocyclic diphosphanes, [{(P*X*_2_)(9,10-η^2^-An)}_2_], with *X* = Cl (Velian *et al.*, 2014 ▸) and phenyl (Riu *et al.*, 2020 ▸). Indeed, the oldest example, now known as the photodimer of anthracene, di-*para*-anthracene, was first reported nearly 160 years ago (see Bouas-Laurent *et al.*, 2000 ▸) and forms as poorly soluble microcrystals when solutions of anthracene in benzene, toluene, *etc*., are exposed to sunlight/UV radiation. Structural characterization by SCXRD showed that the labile dimer arises by symmetrical coupling of the 9,10-carbons on two an­thracenes, where the bridgehead C—C distance [1.624 (3) Å] is long (Choi & Marinkas, 1980 ▸), resulting in a low dissociation energy of the dimer, calculated to be 9 ± 3 kcal mol^−1^ (Grimme *et al.*, 2006 ▸). Inter­estingly, **1** may be considered formally to be a mol­ecule in which atomic copper anions have been inserted into each of the two bridgehead C—C bonds of the anthracene dimer, thereby increasing the separation of the bridgehead C atoms from 1.624 (3) to 3.98 (1) Å.

Unperturbed mononuclear homo-diorganocuprate com­plexes, [Cu*R*_2_]^1−^ (*R* = alkyl, aryl, *etc*.), also have close to linear C—Cu—C units and are formulated to con­tain two carbanions bound to Cu^I^ by quite polar Cu—C σ bonds, where the Cu—C distance in **1** [1.989 (2) Å] is well within the range of 1.83–2.05 Å observed previously for homoleptic diorganocuprates (Davies, 2011 ▸). Although Cu^I^ commonly adopts coordination numbers 2–4 in mononuclear com­plexes, organodicuprate **1** con­tains two quite bulky dianionic hydro­carbyl units, which appear to favor the formation of the observed structure con­taining two-coordinated Cu^I^ with essentially linear C—Cu—C units, as expected for *d* ^10^ Cu^I^ (Cotton *et al.*, 1999 ▸). Owing to the latter, the separation of the 9,10-carbons on individual anthracene groups in **1** effectively defines the Cu⋯Cu distance of 2.6172 (7) Å. Prior studies suggest that *d* ^10^Cu–*d* ^10^Cu inter­actions are very weak to non-existent when the Cu⋯Cu separation is greater than ∼2.50 Å in mol­ecular entities con­taining Cu^I^ com­plexes (Mehrotra & Hoffmann, 1978 ▸; Cotton *et al.*, 1988 ▸; Merz & Hoffmann, 1988 ▸).

Although *d* ^10^Cu–*d* ^10^Cu or ‘cuprophilic’ inter­actions (Harisomayajula *et al.*, 2019 ▸) appear unlikely to stabilize the dimeric structure adopted by **1** in the solid state, the significant contact ion-pairing and resulting increase in its crystalline lattice stabilization may play a key role in the formation of the observed bis­(anthracene)dicuprate(2−) com­plex, relative to unknown monomeric monoanions, *i.e.* [{Cu(THF)_*x*_}(9,10-η^2^-An)]^1−^ (*x* = 0–2), which would likely be strained metallacycles, *vide infra*. In the case of the structurally related organoaluminium com­plexes **2** and **3**, it was proposed that the dimeric, rather than plausible monomeric structures, ‘are favored by smaller deviations from tetra­hedral angles about the aluminum atoms in the dianions rather than about the aluminum atom in the hypothetical monoanions.’ However, surprisingly, the possible importance of contact ion-pairing in contributing to the stabilization of the dimers in the solid state, and possibly also in solution, was not considered (Brauer & Stucky, 1972 ▸). Most inter­estingly, the bis­(naph­tha­lene)­di­ala­nate(2−) salt, **2**, was originally prepared and proposed to be a monomer, [{AlMe_2_}(1,4-η^2^-Np)]^1−^ (Np = naph­tha­lene), based only on a proton NMR spectrum in THF and identification of 1,4-di­hydro­naph­tha­lene as a key hydrolysis product (Lehmkuhl, 1966 ▸). Later, the identical salt, as crystalline **2**, dissolved in THF, was found to afford a proton NMR spectrum in good agreement with that of the purported monomer (Brauer & Stucky, 1970 ▸). In both SCXRD studies on **2** and **3**, the possibility that they could be present as monomers in solution appears to have been implicitly rejected (Brauer & Stucky, 1970 ▸, 1972 ▸). However, the later structural authentication of an (anthracene)aluminium monomer, the neutral metallacycle [{AlEt(THF)}(9,10-η^2^-9,10-bis­(tri­methyl­sil­yl)anthracene)], **4**, strongly suggests that the natures of **2** and **3** in solution merit reinvestigation. It should also be emphasized that presently we cannot rule out the possibility that monomeric forms of **1** could be present in THF or other solvents (see Section 2).

The structure of the alane monomer **4** is consistent with that expected of a strained metallacycle, *vide infra*, *i.e.* a sharp C9—Al—C10 angle of 81.5° for four-coordinated aluminium and an average Al—C(bridgehead) distance of 2.057 (4) Å, which is significantly longer than the normal Al—C(Et) distance of 1.966 (4) Å present in **4** (Lehmkuhl *et al.*, 1985 ▸). Structurally similar (anthracene)alane and (anthracene)alan­ate(1−) monomers have been prepared recently by novel additions of anthracene to an Al^I^ com­plex (Bakewell *et al.*, 2020 ▸) and a di­alkyl­aluminyl anion (Sugita *et al.*, 2020 ▸), respectively.

A more well-known related com­plex is the monomeric (anthracene)Mg solvate in crystalline [{Mg(THF)_3_}(9,10-η^2^-An)], **5**, possessing a C9—Mg—C10 angle of 71.4°. This angle is significantly sharper than the corresponding angle in **4**, likely owing in part to the higher coordination number of Mg in **5**. The latter also con­tains a rather long Mg—C distance of 2.30 (2) Å (Engelhardt *et al.*, 1988 ▸) com­pared to that present in [(9-anthracen­yl)Mg(μ-Br)·di-*n*-butyl ether]_2_, a dimeric Grignard reagent with bridging bromides and an essentially unstrained Mg—C bond with a distance of 2.132 (2) Å (Bock *et al.*, 1996 ▸). For related reasons, **5** appears to be the first (anthracene)metal com­plex to be recognized as a strained metallacycle (Bogdanović *et al.*, 1987 ▸). In solution, **5** is a metastable species, which following facile loss of THF in hexane or diethyl ether, readily eliminates free anthracene and is proposed to initially form ‘quasi-atomic’ magnesium under strictly anaerobic conditions (Bönnemann *et al.*, 1983 ▸). The latter rapidly forms a mirror of magnesium metal in the absence of other reactants (Alonso *et al.*, 1987 ▸). Compound **5** has been found by the Cummins group (Velian & Cummins, 2012 ▸) and others to be a highly useful precursor to numerous new strained monomeric main group element adducts of anthracene, including the formal Ge^IV^ com­plex [{GeMe_2_}(9,10-η^2^-An)], **6**, or 2,3:5,6-dibenzo-7-di­methyl­ger­mano­rbor­na­diene. X-ray structure characterization of **6**, Me_2_GeAn, revealed a sharp C9—Ge—C10 angle of 77.72 (5)°, and Ge—C(9,10 or bridgehead) distances [average 2.030 (1) Å] which are significantly longer than the Ge—C(Me) distance [average 1.943 (4) Å] (Velian *et al.*, 2015 ▸). The latter is statistically identical to that observed for Me_4_Ge [1.945 (3) Å; Hencher & Mustoe, 1975 ▸]. Compound **6** suffers thermal loss of anthracene at 373 K in toluene to produce in good yield an intriguing di­methyl­germylene, Me_2_Ge, adduct [{Ge_2_Me_4_}(9,10-η^2^-An)], **7**, which may arise *via* insertion of highly reactive Me_2_Ge into a strained Ge—C(bridgehead) bond of **6**. However, also possible is an initial dimerization of Me_2_Ge to the digermene, Me_4_Ge_2_, followed by its facile [4 + 2] cyclo­addition to free anthracene, which was established previously to afford **7** (Sakurai *et al.*, 1982 ▸). Also noteworthy is that the thermolysis of two equivalents of **6** to form the less strained **7** and free anthracene has been calculated to have a quite favorable free energy change (Δ*G*) of *ca* −37 kcal mol^−1^ of **7** (Velian *et al.*, 2015 ▸). Finally, under milder conditions, **6** has been shown to function as an Me_2_Ge group transfer reagent (Geeson *et al.*, 2019 ▸).

Of particular relevance to our discussion is that neutral **6** is isoelectronic and likely of similar structure to the presently unknown alanate, [{AlMe_2_}(9,10-η^2^-An)]^1−^, the monomer of structurally characterized dimeric **3** (Brauer & Stucky, 1972 ▸). To the best of our knowledge, none of the reported formally strained metallacyclic monomers, [{*ML_n_*}(9,10-η^2^-An)]^*z*^ (*z* = 0 or −1), are presently known to form dimers (or oligomers) related to **1**, **2**, or **3**. Calculations on the structures and relative stabilities of the monomeric forms of **1**, **2**, and **3**, com­pared to the respective dimers, would be of considerable inter­est. Inclu­sion of contact ion-pairing could be of key importance in such a study, but might prove to be a nontrivial extension.

In contrast to **1**, which con­tains only Cu—C σ-bonds, all prior (arene)copper com­pounds have been characterized as π-com­plexes, including the homoleptic cationic species, [Cu(arene)_2_]^1+^ [arene = 1,2-di­fluoro­benzene (Santiso-Quiñones *et al.*, 2009 ▸), 1,3,5-tri­methyl­benzene, and hexa­methyl­benzene (Wright *et al.*, 2010 ▸, 2015 ▸)], which exhibit the long-known copper–η^2^-arene structural motif, first observed in [Cu(benzene)][(μ-Cl)_3_AlCl] (Turner & Amma, 1966 ▸). More recently, unprecedented copper–arene π-com­plexes con­taining unsupported η^6^-arene binding modes in the 18-electron species [Cu(C_6_Me_6_)(P*R*_3_)]PF_6_ (*R* = phenyl or phenoxide) were described (Wright *et al.*, 2015 ▸). Although unsupported naph­tha­lene, anthracene, or related polyarene com­plexes of copper remain unknown, except in the case of **1** for anthracene, polyarenes bearing substituents which effectively bind to Cu^I^ can also coordinate to copper. For example, a cationic Cu–η^2^-naph­thyl com­plex with a neutral 1-naph­thyl-appended NS_2_ macrocyclic ligand (Conry, 1998 ▸) and an un­usual [Cu{η^6^-9,10-bis­(*N*-*n*-propyl-*N*-di­phenyl­phosphano)am­ino­meth­yl)anthracene}]^1+^ com­plex, in which Cu^I^ is con­strained *via* chelation with di­phenyl­phosphanyl groups to lie over the central ring of nearly planar anthracene (Xu *et al.*, 2003 ▸), have been characterized by SCXRD. Whereas the Cu–η^2^-naph­thyl com­plex has unexceptional Cu—C distances [2.129 (6) and 2.414 (6) Å] for asymmetric π-bonding of the 1-naph­thyl group, the Cu—C distances in the copper–η^6^-anthracene com­plex are in the range 2.773–3.021 Å. These are much longer than usual in (arene)copper com­plexes, indicative, at best, of a very weak copper–anthracene inter­action (Xu *et al.*, 2003 ▸). For example, in [Cu(η^6^-C_6_Me_6_)(P*R*_3_)]PF_6_, the Cu—C distances are in the range 2.253–2.300 Å (Wright *et al.*, 2015 ▸).

## Experimental

### Synthesis and crystallization

All manipulations were carried out under argon in a standard glove-box and/or using Schlenk techniques to maintain strictly anaerobic conditions (Shriver, 1969 ▸; Wayda & Darens­­bourg, 1987 ▸). Solvents were dried by standard methods, as described previously (Brennessel & Ellis, 2012 ▸). Reagent-grade anthracene (99%) was sublimed *in vacuo* and [CuCl(PCy_3_)]_2_ (Cy = cyclo­hex­yl) was prepared as described previously (Churchill & Rotella, 1979 ▸). NMR samples were sealed under argon into 5 mm tubes and analyzed on a Varian Unity 500 MHz or a Bruker Avance III 400 MHz spectrometer. ^1^H and ^13^C chemical shifts are given with reference to residual ^1^H and ^13^C solvent resonances relative to tetra­methyl­silane.

#### [K(THF)_2_]_2_[{Cu(C_14_H_10_)}_2_] (1)

Sublimed anthracene (0.939 g, 5.27 mmol) and shiny pieces of potassium metal (0.211 g, 5.40 mmol) were transferred in an argon-filled glove-box to a round-bottomed Schlenk flask, along with a glass-enclosed magnetic stirrer bar. Subsequently, THF (100 ml) was added and the mixture was stirred vigorously in the dark for 6 h at 293 K to afford a deep-blue solution of potassium anthracene (KAn), which is susceptible to photo-oxidation by visible light. This solution/slurry was cooled to 200 K with stirring and to it was transferred *via* cannula a cold (200 K) colorless solution of [CuCl(PCy_3_)]_2_ (1.000 g, 1.318 mmol) in THF (50 ml) and stirring continued for 12 h at 200 K. The resulting yellow–brown solution was warmed over a *ca* 6 h period to near 290 K (room tem­per­a­ture) and filtered (medium or P4 porosity frit) to remove KCl. After careful evaporation of all but about 20 ml of solvent *in vacuo* at 273–293 K, pentane (100 ml) was added with stirring. The resulting slurry was filtered, washed vigorously with pentane (2 × 10 ml) and dried *in vacuo* to afford 0.59 g (53% yield, based on the copper precursor) of homogeneous yellow solid [K(THF)_2_]_2_[{Cu(C_14_H_10_)}_2_], **1**. No elemental analysis was con­ducted on the bulk solid **1**, so its com­position is based exclusively on the SCXRD study and NMR spectra. X-ray-quality yellow blocks of **1** were grown from a pentane-layered saturated solution in THF at 240 K over a 6 d period.

^1^H NMR (500 MHz, 293 K, THF-*d*_8_, δ, ppm): 1.77 (*m*, 4H, THF), 3.52 (*s*, 1H, H9), 3.63 (*m*, 4H, THF), 6.24 (*m*, 2H, H1), 6.35 (*m*, 2H, H2). ^13^C{^1^H} NMR (125 MHz, 293 K, THF-*d*_8_, δ, ppm): 24.2 (*m*, THF), 52.4 (*s*, C9), 66.3 (*m*, THF), 117.7 (*s*, C1), 117.9 (*s*, C2), 145.2 (*s*, C11).

It is inter­esting that the first structurally authenticated (by SCXRD) monomeric (anthracene)metal com­plex, [{Mg(THF)_3_}(9,10-η^2^-An)], **5** (Engelhardt *et al.*, 1988 ▸), was originally formulated to be a monomer based only on its NMR spectra in THF. Noteworthy is that the latter spectra, without THF, exhibit ^1^H and ^13^C{^1^H} resonances for the coordinated anthracene in **5** with quite similar values to those observed for **1**; *i.e.* ^1^H NMR (400 MHz, 293 K, THF-*d*_8_, δ, ppm): 3.51 (*s*, 1H, H9), 5.95 (*m*, 2H, H1), 6.01 (*m*, 2H, H2); ^13^C{^1^H} NMR (75.4 MHz, 293 K, THF-*d*_8_, δ, ppm): 57.7 (*s*, C9), 114.1 (*s*, C1), 118.1 (*s*, C2), 145.9 (*s*, C11) (Bogdanović *et al.*, 1984 ▸). How­ever, based on available data, it would now be premature to suggest that **1** may also be present as a monomer in THF. Noteworthy is that the corresponding NMR spectra for the aluminium dimer **3** in THF apparently have not been reported for com­parison. Also, studies of **1** in other solvents would be of inter­est, as well as further NMR spectral analyses of this cuprate. Unfortunately, this latter work must be carried out independently in another laboratory because we are no longer able to examine this intriguing species.

### Refinement

Crystal data, data collection and structure refinement details are summarized in Table 1 ▸. H atoms on anthracene C atoms were found from difference Fourier maps and refined freely. H atoms on the tetrahydrofuran ligands were placed geometrically and treated as riding atoms, with C—H = 0.99 Å and *U*_iso_(H) = 1.2*U*_eq_(C).

## Results and discussion

The asymmetric unit of **1**, which is one-half of the formula unit [K(THF)_2_]_2_[{Cu(9,10-η^2^-An)}_2_], con­tains one copper center and one anthracene ligand in contact with one [K(THF)_2_]^1+^ counter-ion. A crystallographic inversion center generates full well-resolved **1** (Fig. 1 ▸). The potassium cations have normal ligated THF with K—O distances [average 2.74 (7) Å] within the range 2.62 (2)–2.78 (3) Å of those observed previously in [K(THF)_2_]_2_[*ML_n_*], where *ML_n_* is [V(η^4^-Np)(η^6^-Np)]^2−^ (Np = naph­tha­lene; Kucera *et al.*, 2022 ▸) and [U(NHDipp)_5_]^2−^ (Dipp = 2,6-diiso­propyl­phenyl; Nelson *et al.*, 1992 ▸). To help understand the mol­ecular structure of the contact ion-pair com­plex, **1**, it is useful to first com­pare details of the metal–metal inter­actions in **1** with those in the only previously known (anthracene)metal dimer com­plex present in [Na(THF)_2_]_2_[{(AlMe_2_)(9,10-η^2^-An)}_2_], **3**, (Brauer & Stucky, 1972 ▸). It is important to recognize that both metal atoms, K and Cu, in **1** are appreciably larger than the corresponding atoms, Na and Al, in **3**. Thus, the sum of the covalent radii of K and Cu (3.35 Å) is greater than the corresponding sum for Na and Al (2.87 Å) (Cordero *et al.*, 2008 ▸). However, the formally nonbonded distances (Table 2 ▸) between all of the metals in **1** are considerably shorter, *i.e.* K1⋯Cu1 = 3.6637 (15) Å, K1⋯Cu1*A* = 3.3762 (9) Å, K1⋯K1*A* = 6.542 (2) Å and Cu1⋯Cu1*A* = 2.6171 (7) Å [symmetry code: (*A*) −*x*, −*y* + 1, −*z* + 1], than the corresponding distances in **3**, *i.e.* Na1⋯Al1 = 4.348 (6) Å, Na1⋯Al1*A* = 4.455 (7) Å, Na1⋯Na1*A* = 7.28 (1) Å and Al1⋯Al1*A* = 4.95 Å. As described in Section 1, the rather short, but formally nonbonded, Cu⋯Cu distance in **1** arises from the essentially linear C9—Cu—C10*A* angle, expected for two-coordinated *d* ^10^ Cu^I^ (Cotton *et al.*, 1999 ▸).

However, in **3**, the considerably smaller C9—Al—C10*A* angle [120.2 (4)°], resulting from the distorted tetra­hedral geometry of four-coordinated Al^III^, causes the Al⋯Al distance to be much longer (Fig. 2 ▸). Of particular inter­est is that the relatively uncrowded linear Cu atoms in **1** permit both solvated potassium ions to approach the cuprate centers more closely than the smaller solvated sodium ions can with the bulkier distorted tetra­hedral aluminate centers in **3**. Whereas the Na⋯Al distances in **3** exhibit a small difference [0.107 (7) Å] and, as a result, its mol­ecular structure, without the THF groups, is fairly symmetrical and deviates only slightly from *D*_2*h*_ symmetry (Brauer & Stucky, 1972 ▸), the two K⋯Cu distances in **1** differ by 0.287 (2) Å (Fig. 1 ▸), with one potassium ion having a surprisingly short K⋯Cu distance of 3.3762 (9) Å, close to the sum of the covalent radii of K and Cu (3.35 Å) (Cordero *et al.*, 2008 ▸). As a consequence, **1** differs from **3** in having a slightly more com­pact but a much less symmetrical contact ion-pair structure, owing largely to the different coordination numbers of copper and aluminium, in otherwise quite similar species. Another key difference between **1** and **3** are the nonbonded bridgehead C9⋯C10*A* distances [3.974 (3) and 3.57 (1) Å, respectively], which show that the two formal 9,10-coordinated anthracene dianion units are slightly further apart in **1** than they are in **3**, owing also to the different coordination numbers of copper and aluminium in these remarkable com­pounds, both of which are worthy of additional study.

Despite the aforementioned differences, the geometries of the central anthracene ring of **1** and **3** are similar. Thus, for **1**, the bridgehead angles C11—C10—C14 [113.3 (2)°] and C12—C9—C13 [113.1 (2)°] (Table 2 ▸) do not differ significantly from the corresponding angles reported for **3** [114.1 (9) and 112 (1)°; Brauer & Stucky, 1972 ▸]. Similarly, the bridgehead C—C distances for **1** [C9—C12 = 1.482 (3) Å, C9—C13 = 1.480 (3) Å, C10—C11 = 1.479 (3) Å, and C10—C14 = 1.478 (3) Å, with average C—C = 1.480 (3) Å; Table 2 ▸], are statistically identical to corresponding C—C distances reported for **3** [average 1.49 (2) Å], which are both close to the value of 1.51 (5) Å observed previously for C(*sp* ^2^)—C(*sp* ^3^) distances (Jolly, 1976 ▸). Perhaps surprisingly, the geometry of the coordinated central ring of anthracene in the first reported structurally characterized anionic metallacyclic monomer of this type, [{Lu(η^5^-Cp)_2_}(9,10-η^2^-An)]^1−^, is structurally nearly identical, without the metal, to those observed in the dianions of **1** and **3**, with an average bridgehead C—C distance of 1.485 (9) Å. The lutetium anion also has an average bridgehead C—C—C angle of 111.8 (5) Å, which is very close to the corresponding angles of **1** and **3**. A key structural difference in the lutetium anion is the sharp C9—Lu—C10 angle of 67.1 (2)°, owing to the metalla­cyclo­propane character of the com­plex and, likely also the large bulk of the bis­(cyclo­penta­dien­yl)lutetium moiety, which may well be responsible for its stability towards dimerization or oligomerization in solution (THF) and in the solid state as a crystalline [Na(diglyme)_2_]^1+^ salt (diglyme = di­ethyl­ene glycol dimethyl ether) at *ca* 293 K (Roitershtein *et al.*, 1993 ▸).

Inter­actions of the alkali metal cations with the *exo*-benzene C atoms are consistent with the overall mol­ecular structures of **1** and **3**. In the less symmetrical cuprate **1**, the K⋯C distances fall into a distinct 1:1 pattern, with the six C atom closest to the short K⋯Cu contact ion pairing (C1, C2, C5, C6, C11, and C13) having the shortest distances [average 3.14 (13) Å], whereas the K⋯C distances for the six C atoms closest to the long K⋯Cu contact (C3, C4, C7, C8, C12, and C14) are longer [average 3.38 (5) Å] (see Figs. 1 ▸ and 3 ▸). In contrast, for the aluminate **3**, the Na—C distances for the eight outer C atoms are rather similar and range from 2.94 (1) to 3.24 (1) Å, but the Na—C distances of the four more crowded ring-junction C atoms are mostly longer [average 3.28 (4) Å; Brauer & Stucky, 1972 ▸]. Finally, in **1**, the essentially planar *exo*-benzene units possess C—C distances [average 1.396 (3) Å] and C—C—C angles [average 119.9 (2)°] that are quite similar to those found in uncharged free benzene (Mitchell & Cross, 1965 ▸), indicating that the contact ion pairing is sufficiently weak to have no significant influence on the structure of the *exo*-benzene groups and that the negative charge on each anthracene ligand is essentially localized on the central ring and the appended metal center. Similar features were found for the *exo*-benzene groups in **3** (Brauer & Stucky, 1972 ▸). However, in both **1** and **3**, the inter­actions of the counter-ions in these contact ion-paired com­plexes may play an important role in defining the structures of the anionic com­ponents and perhaps their existence in the solid state. In this respect, the isolation of the bis­(anthracene)dicuprate(2−) with relatively weakly inter­acting cations, such as [K([2.2.2.]cryptand)]^1+^, tetra­alkyl­ammonium­(1+), *etc*., would be of considerable inter­est. As our laboratory will be irreversibly shuttered in 2023, others are encouraged to examine these possibilities and related issues, *vide infra*.

Since our initial report on tris­(1,2,3,4-η^4^-naph­tha­lene)­zir­co­nate(2−), the first confirmed homoleptic (polyarene)metallate of a *d*-block metal (Jang & Ellis, 1994 ▸), which was remarkably labile and functioned formally as a ‘masked’ naked source of low-spin atomic Zr^2−^ in its facile reaction with carbon monoxide to afford [Zr(CO)_6_]^2−^, our research group has been inter­ested in discovering what other *d*-block elements, throughout the periodic table, would afford related and hopefully labile com­plexes. Thus began our exploration in a systematic fashion of the synthesis and reactivity patterns of transition-metal com­pounds con­taining metals in formal negative oxidation states (Ellis, 2006 ▸). Early on we wondered whether *f*-block elements would also ‘succumb’ to this strategy, but unfortunately never examined these elements (Ellis, 2019 ▸). In this regard, we would like to point to the exciting recent results of Skye Fortier and co-workers in highly challenging uranium chemistry (Murillo *et al.*, 2021 ▸, 2022 ▸). As mentioned in Section 1, we were also intrigued by early reports of ‘anionic copper’ (Rieke *et al.*, 1990 ▸) and this led to our synthesis and structural characterization of the totally unexpected dicuprate salt, **1**, described herein. Owing to the early departure of a group member, extension of this study was not possible. For example, we had hoped to examine the reactivity of **1** with good acceptor ligands, such as CO, PF_3_, and P(O*R*)_3_, and particularly organic isocyanides, to determine whether the anthracenes would be displaced to produce new formal Cu(0,1−) com­plexes. Noteworthy is that although isolable Cu^1−^ com­plexes remain unknown, very recently, the first unambiguous Cu^0^ com­plex was isolated, thoroughly characterized, and structurally authenticated (Graziano *et al.*, 2022 ▸). Also, examination of Rieke’s thermally unstable alleged cuprates, derived from naph­tha­lene radical anion reductions remain of great inter­est because the possible ‘naph­tha­lene stabilized cuprate’ may be a more labile source of ‘naked Cu(1−)’ than one derived from the anthracene radical anion. Extension of these studies to silver and gold promise to uncover exciting results. For example, could a *bona fide* gold anion (Jansen, 2008 ▸) be ‘tamed’ by naph­tha­lene or anthracene to provide labile com­plexes, enabling the study of new aurate chemistry? We mention these possible extensions because none of this research will be carried out by us at the University of Minnesota.

## Supplementary Material

Crystal structure: contains datablock(s) I, global. DOI: 10.1107/S2053229623008367/eq3012sup1.cif

Structure factors: contains datablock(s) I. DOI: 10.1107/S2053229623008367/eq3012Isup2.hkl

CCDC reference: 2296812

## Figures and Tables

**Figure 3:**
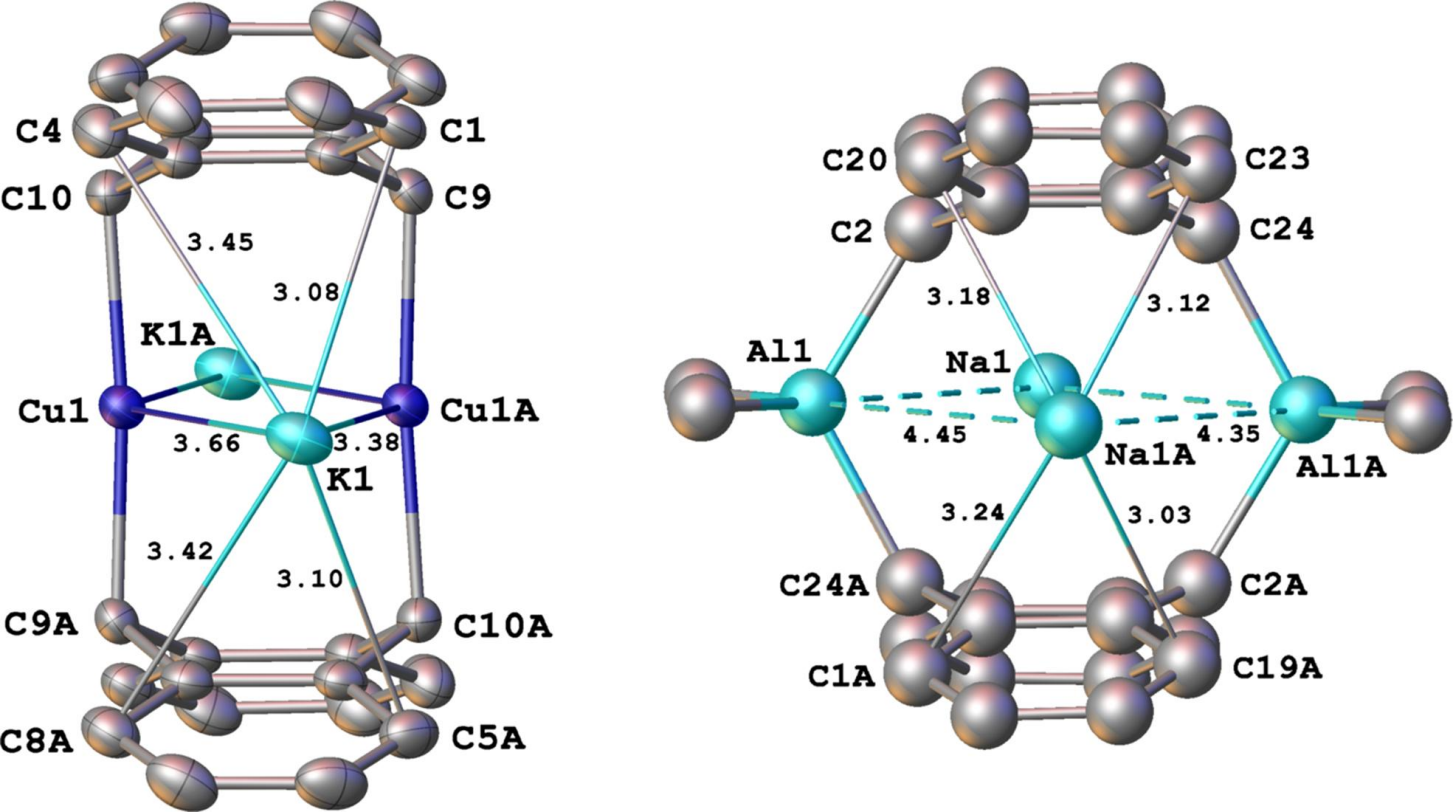
End views of **1** (left) and **3** (right) that highlight the much greater asymmetry of the dicuprate. Representative atom–atom distances are shown (Å). In **1**, the six closest C atoms to the shorter Cu⋯K contact have an average K⋯C distance of 3.14 (13) Å, while those closest to the longer Cu⋯K contact have an average K⋯C distance of 3.38 (5) Å.

**Table 1:** Experimental details

Crystal data	
Chemical formula	[K(C_4_H_8_O)_2_]_2_[Cu(C_14_H_10_)_2_]
*M*_r_	850.13
Crystal system, space group	Triclinic, *P* 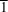
Temperature (K)	173 (2)
*a*, *b*, *c* (Å)	9.6864 (19), 10.484 (2), 10.658 (2)
α, β, γ (°)	66.22 (3), 89.67 (3), 82.73 (3)
*V* (Å^3^)	981.2 (4)
*Z*	1
Radiation type	Mo *K*α
μ (mm^−1^)	1.34
Crystal size (mm)	0.40 × 0.40 × 0.20

Data collection	
Diffractometer	Bruker SMART platform CCD
Absorption correction	Multi-scan (*SADABS*; Sheldrick, 1996 ▸)
*T*_min_, *T*_max_	0.630, 0.746
No. of measured, independent and observed [*I* > 2σ(*I*)] reflections	11455, 4459, 3853
*R*_int_	0.024
(sin θ/λ)_max_ (Å^−1^)	0.650

Refinement	
*R*[*F* ^2^ > 2σ(*F* ^2^)], *wR*(*F* ^2^), *S*	0.029, 0.073, 1.04
No. of reflections	4459
No. of parameters	275
H-atom treatment	H atoms treated by a mixture of independent and constrained refinement
Δρ_max_, Δρ_min_ (e Å^−3^)	0.34, −0.31

Computer programs: *SMART* (Bruker, 2003 ▸), *SAINT* (Bruker, 2003 ▸), *SHELXT2018* (Sheldrick, 2015*a* ▸), *SHELXL2019* (Sheldrick, 2015*b* ▸), and *SHELXTL* (Sheldrick, 2008 ▸).

**Table 2:** Selected geometric parameters (Å, °)

Cu1—C9^i^	1.9873 (19)	C8—K1^i^	3.421 (2)
Cu1—C10	1.9906 (19)	C9—C13	1.480 (3)
Cu1—Cu1^i^	2.6172 (7)	C9—C12	1.482 (3)
Cu1—K1^i^	3.3762 (9)	C10—C14	1.478 (3)
Cu1—K1	3.6637 (15)	C10—C11	1.479 (3)
C1—K1	3.081 (2)	C11—K1^i^	3.158 (2)
C2—K1	3.158 (2)	C12—K1^i^	3.3324 (19)
C3—K1	3.338 (3)	C13—K1	3.170 (2)
C4—K1	3.446 (2)	C14—K1	3.364 (2)
C5—K1^i^	3.100 (2)	K1—O1	2.681 (2)
C6—K1^i^	3.203 (3)	K1—O2	2.8027 (16)
C7—K1^i^	3.360 (2)		
			
C9^i^—Cu1—C10	174.74 (7)	C13—C9—C12	113.07 (15)
C9^i^—Cu1—Cu1^i^	91.87 (6)	C14—C10—C11	113.34 (15)
C10—Cu1—Cu1^i^	93.22 (6)		

Symmetry code: (i) 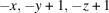 .
